# Development of implantable electrode based on bioresorbable Mg alloy for tissue welding application

**DOI:** 10.1038/s41598-024-67077-8

**Published:** 2024-07-12

**Authors:** Lin Mao, Zhengyi Han, Xupo Xing, Zhongxin Hu, Langlang She, Chengli Song

**Affiliations:** Shanghai Institute for Minimally Invasive Therapy, School of Health Science and Engineering, University of Shanghai for Science and Technology, Shanghai, 200093 China

**Keywords:** Radiofrequency tissue welding, Mg alloy, Implantable electrode, Structural design, Electrothermal simulation, Anastomotic strength, Engineering, Mathematics and computing

## Abstract

An implantable electrode based on bioresorbable Mg-Nd-Zn-Zr alloy was developed for next-generation radiofrequency (RF) tissue welding application, aiming to reduce thermal damage and enhance anastomotic strength. The Mg alloy electrode was designed with different structural features of cylindrical surface (CS) and continuous long ring (LR) in the welding area, and the electrothermal simulations were studied by finite element analysis (FEA). Meanwhile, the temperature variation during tissue welding was monitored and the anastomotic strength of welded tissue was assessed by measuring the avulsion force and burst pressure. FEA results showed that the mean temperature in the welding area and the proportion of necrotic tissue were significantly reduced when applying an alternating current of 110 V for 10 s to the LR electrode. In the experiment of tissue welding ex vivo, the maximum and mean temperatures of tissues welded by the LR electrode were also significantly reduced and the anastomotic strength of welded tissue could be obviously improved. Overall, an ideal welding temperature and anastomotic strength which meet the clinical requirement can be obtained after applying the LR electrode, suggesting that Mg-Nd-Zn-Zr alloy with optimal structure design shows great potential to develop implantable electrode for next-generation RF tissue welding application.

## Introduction

Intestinal structure reconstruction is usually restored by surgical operation after diseased tissue is removed in surgery. The quality of anastomotic stoma and the recovery of intestinal physiological function are important indicators for evaluating the performance of connected tissue. In the past, manual suture is often used for intestinal anastomoses^1,2^. However, it is time-consuming and requires a high level of surgical skill possessed by the operator. After the introduction of laparoscopic surgery, staplers have become the most widely used equipment in clinical for intestinal anastomoses by puncturing and connecting tissues through titanium nails^3^. However, mechanical damage from the anastomotic nail puncture may cause rupture and bleeding of the anastomotic stoma after operation^4^. Furthermore, titanium nails remain in the tissue after surgery, thereby causing several side effects such as foreign body residue and long-term chronic inflammation^5–7^ in the human body.

In recent years, there have been various efforts to explore tissue welding (fusion) technology based on radiofrequency (RF) energy^8,9^. This technology provides an innovative method for structural reconstruction of biological tissues through forming a seamless anastomotic stoma with no foreign body (i.e. suture, staple) residue and mild inflammatory reaction. Smulders et al.^10^ carried out side-to-side anastomoses of intestines based on RF energy using a LigaSure device in a live porcine model, verifying the feasibility of RF-induced intestinal anastomoses by observing the healing process of granulation tissue at the anastomotic stoma. Winter et al.^11,12^ explored the effects of pressure, temperature, and welding time on the quality of intestinal anastomoses. Holmer et al.^13^ studied the connection strength of welded tissue through burst pressure measurement after applying different compression pressures. Zhao et al.^14^ studied the effect of different RF energy outputs on the strength of anastomotic stoma through introducing a device with a function of temperature feedback. And later, Zhao et al.^15^ further studied the effect of different electrode structures on the mechanical and thermal damage of welded tissue induced by RF energy. The aforementioned studies demonstrated the viability of RF tissue welding and delved into key factors influencing welding quality, including pressure, temperature, welding duration, and electrode configurations.

Tissue welding based on RF energy is believed to be the result of simultaneously applied RF current and compression pressure (CP), in which the collagen and elastin within tissue walls are denatured, and the compression pressure applied by the welding electrode causes the denatured protein to form a welding band through cross-linking of peptide chains while the tissue walls are in apposition^8,9^. In addition, there are collagen type III and type V, which stabilize the helical conformation of collagen molecules, resulting in greater temperature stability^16^. Therefore, the strength of welded tissue can be determined by the structure of electrode^17^ and the welding parameters (i.e. compressive pressure, energy power and duration time) to a great extent. The main challenges of tissue welding are summarized as follows:

1. The strength of anastomotic stoma formed by collagen deformation is too low to establish a stable tissue connection;
2. Thermal damage exhibits a negative effect on tissue activity, which affects the process of postoperative repair and ultimately results in the failure of tissue connection;
3. The traditional electrode (i.e., copper electrode), is usually stripped from the anastomotic stoma and taken out of the body after welding operation, easily causing damage to the new anastomotic stoma and further weaken the bonding strength of the connected tissues.

**Figure 1:**
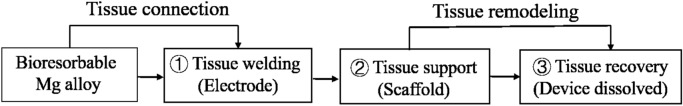
Different functions of Mg-based implantable electrode during the processes of RF tissue welding, tissue support and tissue recovery.

In the light of the unmet clinical needs, bioresorbable magnesium (Mg) alloy is considered as an ideal material to develop implantable electrode with multi-functions for next-generation RF tissue welding application. Firstly, Mg alloy possesses good electrical and thermal conductivity, which can act as a conductor to transmit RF current in tissue welding. Secondly, good processability of Mg alloy makes it easy to fabricate into a scaffold-type electrode to support and strengthen anastomotic stoma after tissue welding. More importantly, Mg alloys possess a natural ability to biodegrade due to corrosion when placed within the human body, and the released corrosion products can be absorbed or metabolized by the body. The functions of the Mg-based device in different stages of tissue reconstruction are shown in Fig. 1. The corrosion of Mg alloys in the physical environment is actually determined by the electrochemical reaction between Mg and water^18^:

$$ \begin{gathered} {\text{anodic reaction}}:{\text{ Mg }} \to {\text{ Mg}}^{{{2} + }} + {\text{ 2e}} \hfill \\ {\text{cathodic reaction}}:{\text{ 2H}}_{{2}} {\text{O }} + {\text{ 2e }} \to {\text{ H}}_{{2}} + {\text{ 2OH}}^{ - } \hfill \\ \end{gathered} $$

The overall reaction can be expressed as follow:

$$ {\text{Mg }} + {\text{ 2H}}_{{2}} {\text{O }} \to {\text{Mg }}\left( {{\text{OH}}} \right)_{{2}} + {\text{ H}}_{{2}} $$

Mg (OH)_2_ is generally insoluble in water. However, the body fluid is rich of acid ions (i.e., CO_3_^2-^, PO_4_^3-^ and Cl^-^), the existence of Cl^-^ could transform insoluble Mg(OH)_2_ into soluble MgCl_2_, then the chemical reaction carries out by the following equation:

$$ {\text{Mg }}\left( {{\text{OH}}} \right)_{{2}} + {\text{2Cl}}^{ - } \to {\text{ MgCl}}_{{2}} + {\text{ 2OH}}^{ - } $$

Therefore, Mg-based implant can disappear completely in the human body after its period of use, leaving no foreign body residue and eliminating the need for a second operation to remove the implant.

In this study, bioresorbable Mg-Nd-Zn-Zr alloy was adopted to develop as an implantable electrode with multi-functions for next-generation RF tissue welding application, and two different structures of the electrodes with the features of cylindrical surface (CS) and continuous long ring (LR) in the welding area were designed. The effect of different structures on temperature distribution, anastomotic strength and histopathologic change of anastomotic stoma were investigated through finite element analysis (FEA) and experiment of tissue welding ex vivo, aiming to verify the feasibility of implantable electrode based on Mg-Nd-Zn-Zr alloy for RF tissue welding application in terms of reducing thermal damage and enhancing tissue anastomotic strength. However, this is just the beginning of implantable Mg-based tissue welding electrode era, and improvements are still needed for the bioresorbable electrode in order to advance to the next stage of matching the corrosion rate in accordance with the tissue growth rate.

## Materials and methods

### Tissue welding models based on implantable electrodes

**Figure 2:**
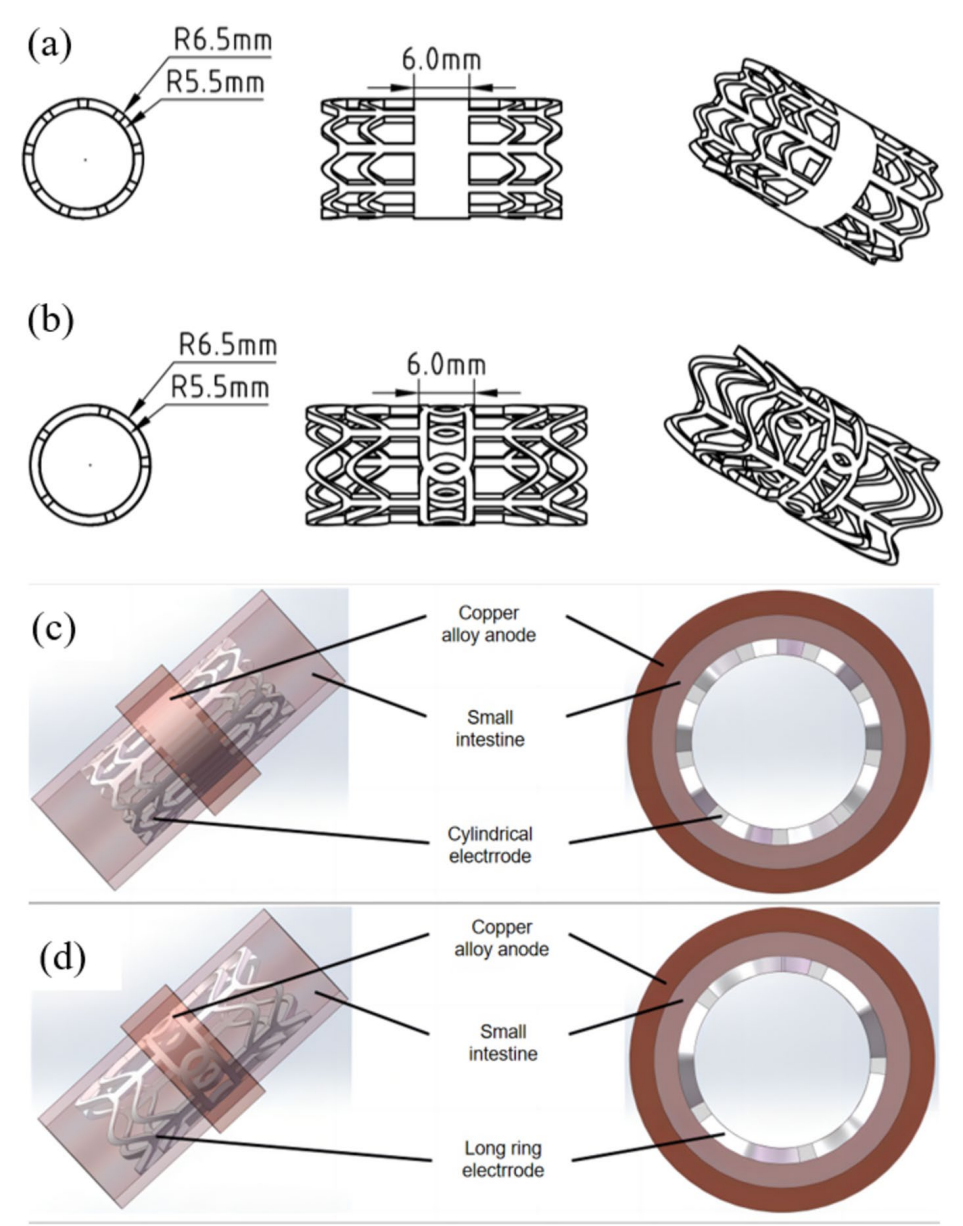
Electrode designs and models of tissue welding. (**a**,**b**) Physical drawings of the two Mg-based electrodes: the top one is the CS electrode and the bottom one is the LR electrode; (**c**,**d**) Tissue welding models established based on the CS and LR electrodes, respectively.

Two pairs of electrodes were designed to create tissue welding models, in which the tissues were aligned in a mucosal-serosa manner between the inner and outer electrodes, as shown in Fig. 2. In both models, the outer electrodes are made of copper alloy, and the inner electrodes with the structural features of CS and LR in the welded area are made of Mg-Nd-Zn-Zr alloy, which possesses excellent biocompatibility^19,20^ and uniform degradation property in the physical environment^18^.

### Pennes bio‐heat transfer equation

Due to the involvement of biological tissues in the model, the widely used Pennies biological heat transfer model was adopted^21^, which takes into account the influence of liquids in the organization on heat conduction to obtain more accurate simulation results. The formula is shown in Eq. (1)^22^.

1 $${\rho {c}_{t}\left(\frac{{\partial T}_{t}}{\partial t}\right)-k}_{t}\nabla \cdot \left(\nabla {T}_{t}\right)={\omega }_{b }{\rho }_{b}{C}_{b}\left({T}_{b}-{T}_{t}\right)+{Q}_{met}+{Q}_{i}$$

where, $\rho$ is the density of intestinal tissue, *c*_t_ is the specific heat of intestinal tissue, *k*_t_ is the thermal conductivity of intestinal tissue, *T*_t_ is the temperature of intestinal tissue, *ω*_b_ is the blood perfusion rate, *ρ*_b_ is the density of blood, *C*_b_ is the specific heat of blood, *T*_b_ is the temperature of blood, *Q*_*met*_ is the metabolic heat generation, *Q*_*i*_ is the electric power absorption.

Because the temperature field simulation was performed on isolated intestinal tissue, the convective heat transfer caused by blood perfusion *ω*_b_*ρ*_b_*C*_b_(*T*_*b*_*-T*_*t*_) and metabolic heat *Q*_*met*_ could be ignored.

Tissue thermal damage is calculated by the Arrhenius equation as expressed below:

2 $$\Omega (\tau )={\int }_{0}^{\tau }A{e}^{\frac{{-E}_{a}}{RT}}dt$$

where $\Omega (\tau )$,$\tau$, *A*, and ${E}_{a}$ are the degree of tissue injury, heating time, frequency factor, and activation energy, respectively.

### Definition of the boundary conditions

The electrothermal simulations during tissue welding process were performed using Comsol 5.6 software. An alternating current of 110 V was applied to the outer copper electrode, and zero voltage was applied to the inner Mg alloy electrode. The temperature variations of the intestine were analyzed for a welding period of 10 s. The initial tissue temperature was 37 °C, and the initial boundary temperature was 25 °C. The material properties used in the simulations were shown in Table 1.

**Table 1:** Material properties.

Name	Density, ρ (kg/m^3^)	Conductivity, σ (S/m)	Thermal conductivity, λ (W/m/°C)	Heat capacity C (J/kg/°C)	Relative dielectric constant, ɛ(1)
Magnesium alloy density	1770	6.417e6	105.224	1e3	1
Copper alloy density	8960	5.998e7	400	385	1
Colonic density	1030	0.07	0.493	3.595e3	8.98e3

### Experiment of tissue welding ex vivo

Fresh porcine small intestines were collected from a local slaughter house (IRB approval and written consent was not needed), and phosphate buffer with a pH of 7.2 and a temperature of 0 °C was prepared for storage of the fresh tissues that was previously cleaned with 0.9% normal saline solution. Intestinal tissues were aligned in a mucosal-serosa manner on the Mg based electrode mounted on an insulated and heat-resistant ring carrier.

**Figure 3:**
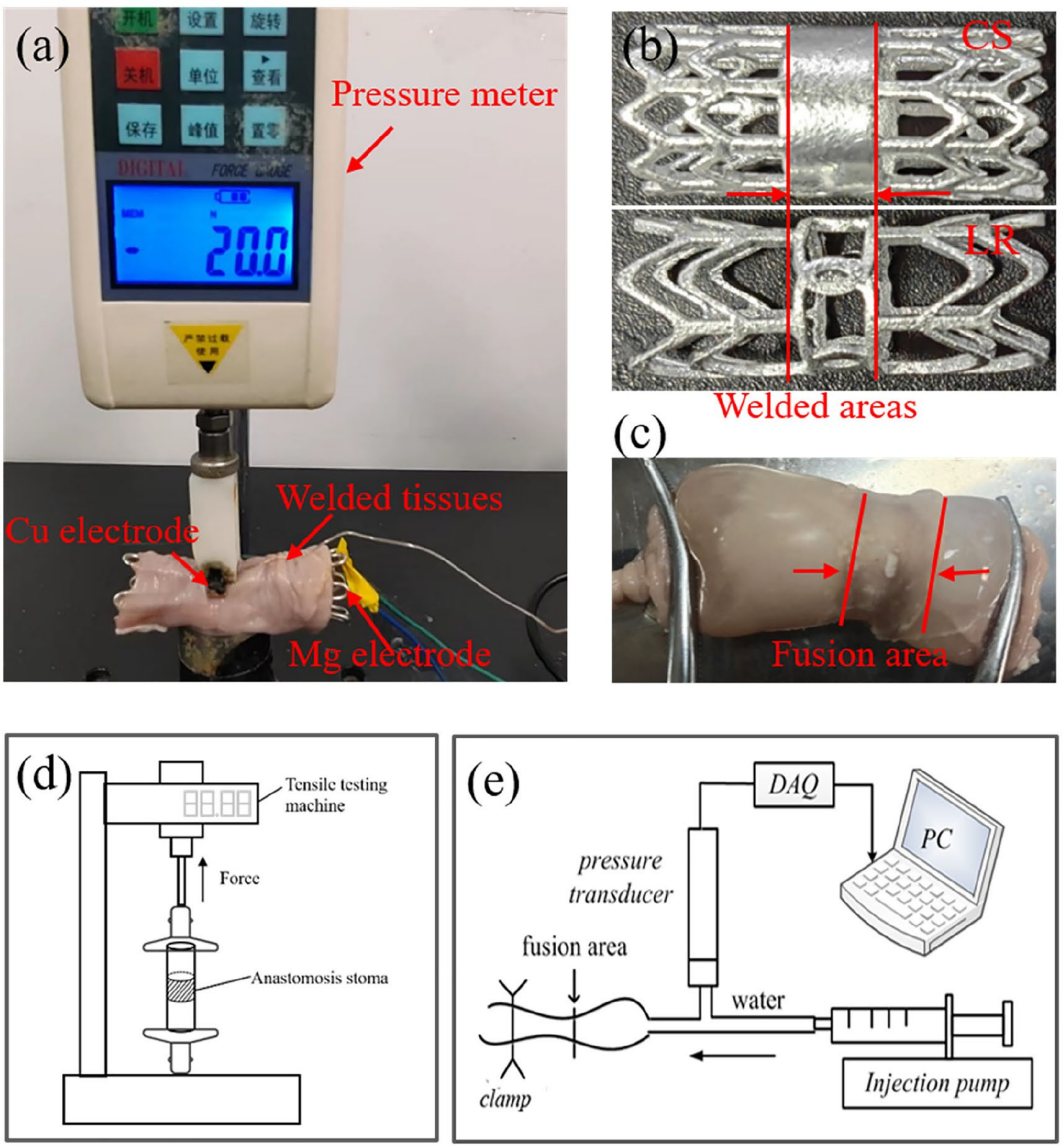
Experiment of tissue welding ex vivo. (**a**) The picture of tissues in RF welding experiment under the proper compressive pressure; (**b**) images of the two Mg-based electrodes; (**c**) typical anastomotic anastomotic stoma welded by the LR electrode; (**d**,**e**) schematic diagrams of the avulsion force and burst pressure measurements.

Bioresorbable electrodes based on Mg-Nd-Zn-Zr alloy with different structures in the welding area were fabricated for RF intestinal tissue welding, as shown in Fig. 3. The main experimental parameters for tissue welding are shown in Table 2. A pressure device that hold the external copper electrode could provide pressure to the target tissue. LigaSure vessel sealing system (Valley lab, Covidien, USA) was used to provide RF energy for tissue welding. Two severed tissues were aligned in mucosa-serous manner and welded under a constant compression pressure of 176 kPa, different RF energy and welding time, and the welding process was shown in Fig. 3a. A close-up of the two electrodes and the typical anastomotic stoma welded by the LR electrode were shown in Fig. 3b,c. Infrared thermal camera was used to monitor the temperature change of the intestines in real-time to verify the effect of both electrodes on temperature. In order to explore the effects of different welding parameters on the biomechanical properties of welded tissues, the avulsion force test and burst pressure measurement of the welded tissues were carried out, and the schematic diagrams of biomechanical performance tests were illustrated in Fig. 3d,e. The parallel experiment was conducted five times.

**Table 2:** Experimental parameters for tissue welding.

Group	Energy power (W)	Welding time (s)
1	120	6
2	140	6
3	160	6
4	120	8
5	140	8
6	160	8
7	120	10
8	140	10
9	160	10

### Strength test of anastomotic stoma

The strength of anastomotic stoma was estimated by the burst pressure and avulsion force testing. The burst pressure is defined as the maximum pressure at which the fusion anastomotic stoma begins to leak during infusion^23,24^. The equipment used for burst pressure measurement in this study mainly includes a T-tube, pressure sensor, constant flow pump, and data acquisition card. When water was pumped into the intestinal lumen at a rate of 2 ml/min^25^, the pressure signal was transmitted to the computer through the sensor and processed by the data acquisition card. The avulsion force is defined as the maximum tensile force when the anastomotic stoma is tear apart with a tensile speed of 10 mm/min during the pulling process^26^.

### Histopathological examination

Intestinal tissues before and after welding were cut into small pieces with an area of 1 cm^2^ and preserved in a neutral formalin buffer. The pathological alterations of tissues were examined using a fluorescence microscopy (DMi8, Leica, Germany) after a standard operating procedure of dehydration, transparency, wax immersion, embedding, slicing, baking, and hematoxylin–eosin (H&E) staining.

### Statistical analysis

The experimental data were analyzed by SPSS (IBM, USA). Since experimental data were not normally distributed, the Mann–Whitney U test was used to compare the differences between the two groups of data, and the Kruskal–Wallis test was used to compare the differences between multiple groups of data, with significance was set at p < 0.05.

## Results

### Electrothermal simulations

**Figure 4:**
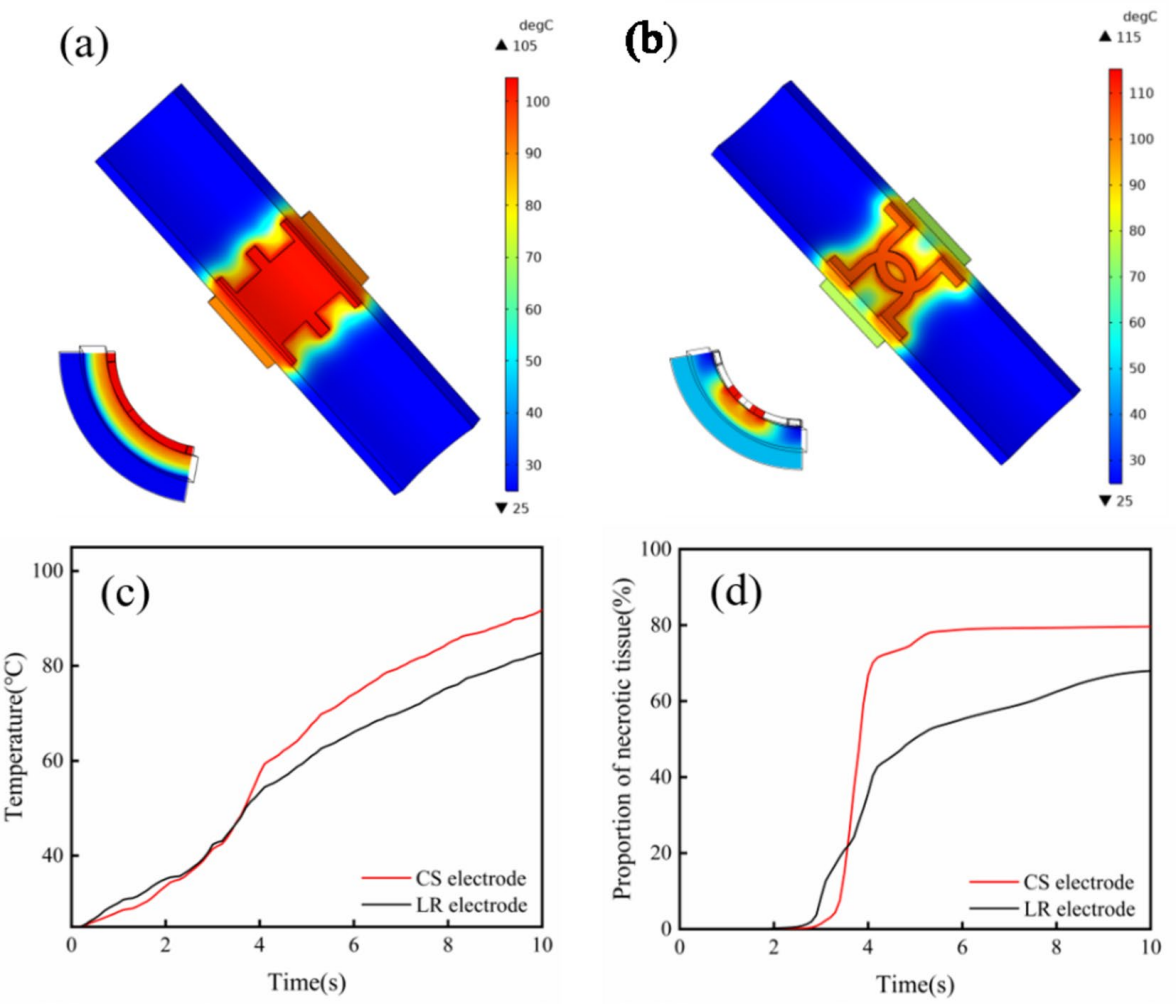
Temperature distributions and proportions of necrotic tissues in electrothermal simulations. Temperature distributions of the models for the CS (**a**) and LR (**b**) electrodes. (**c**) Mean temperatures of welded tissues and (**d**) necrotic tissue proportions obtained by the CS and LR electrodes.

The physical welding model of tissue supported by the implantable Mg-based electrode was established, and a structural element of the welding model was selected to study the electrothermal simulations, as shown in Fig. 4. The highest temperature of the simulation model for the CS electrode was 105 °C, whereas the highest temperature for the LR electrode model was 115 °C. The difference between the two groups could be attributed to the reason that the smaller welding area of the LR electrode formed a larger current density, and thus resulted in a higher welding temperature (Fig. 4a,b). However, from the cross-section of the model, it can be found that the intestinal tissue in contact with the CS electrode showed a higher temperature, while the welded tissue in the hollow area of the LR electrode exhibited a lower temperature. In addition, as shown in Fig. 4c, the mean temperature of tissue welded by the LR electrode (82.8 °C) was lower than that welded by the CS electrode (91.8 °C), and the proportions of necrotic tissues (Fig. 4d) welded by the CS and LR electrodes were 79.6% and 67.9%, respectively, suggesting that a reduced thermal damage was found in the tissue welded by the LR electrode.

**Figure 5:**
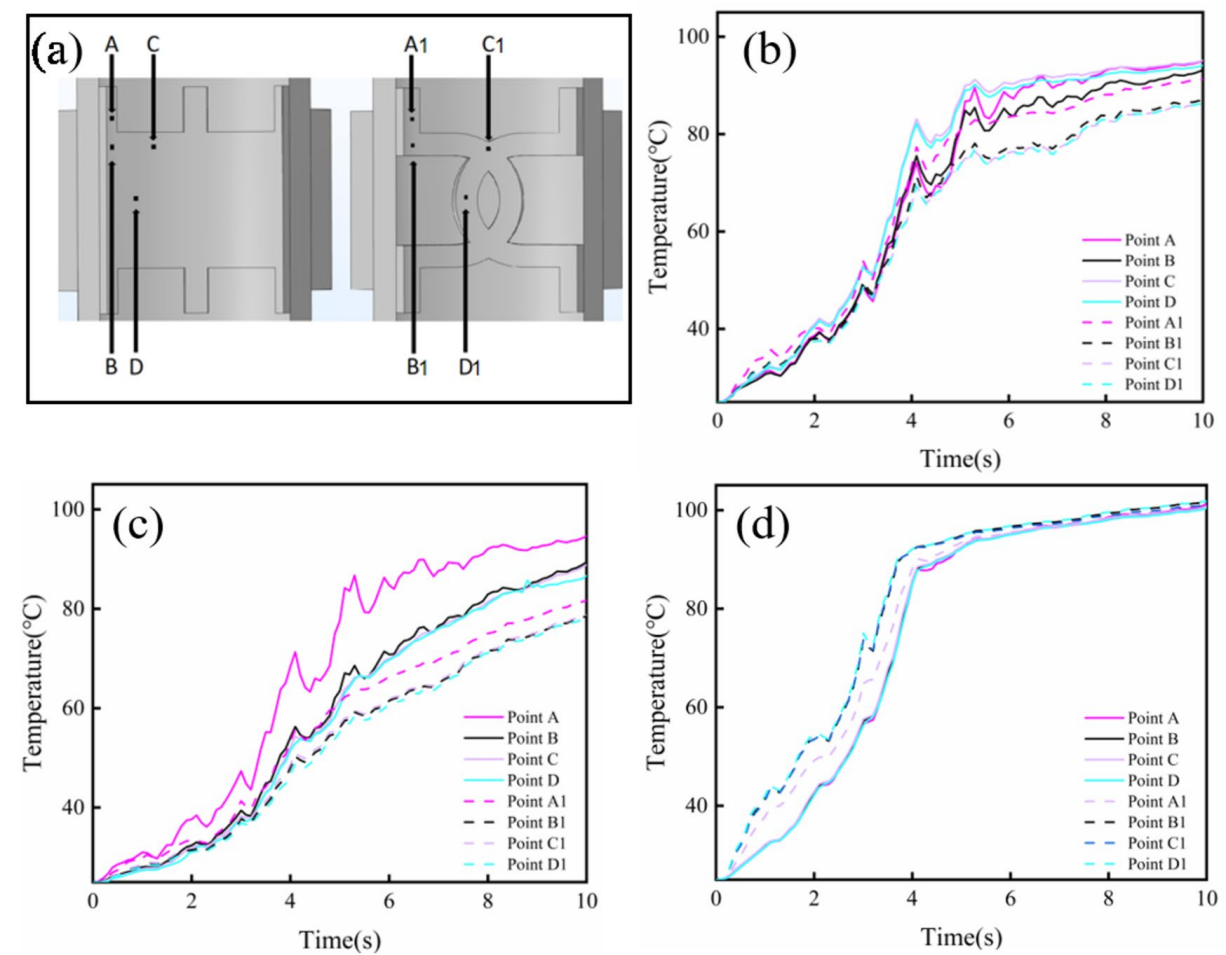
Temperature variations of domain points. (**a**) Domain points on the CS (A-D) and LR (A1-D1) electrodes. Temperature variations of the corresponding positions on the central points of tissues (**b**), the interfaces between the tissue and the outer copper electrodes (**c**) and the interfaces between the tissue and the inner Mg-based electrodes (**d**).

In addition, several domain points which were marked on the CS (A–D) and LR (A1–D1) electrodes were selected for further detailed study of temperature distributions (Fig. 5a). The corresponding position on the central points of tissue, on the interface between the tissue and the outer copper electrode, and on the interface between the tissue and the inner Mg-based electrode were studied to explore the effect of different electrode structure on heat generation and conduction in the simulations. As shown in Fig. 5b, it was obvious that all the corresponding central point temperatures of tissue welded by the LR electrode were substantially lower than that welded by the CS electrode. Similar results were found in the temperature variations of the points on the interface between the tissue and the outer copper electrode, and the temperatures at the edge points of A and A1 was higher than that at the center points of D and D1 for the two groups (Fig. 5c). However, the temperature distributions on the interface between the tissue and the inner Mg-based electrode was relatively similar for the two groups, as shown in Fig. 5d.

**Figure 6:**
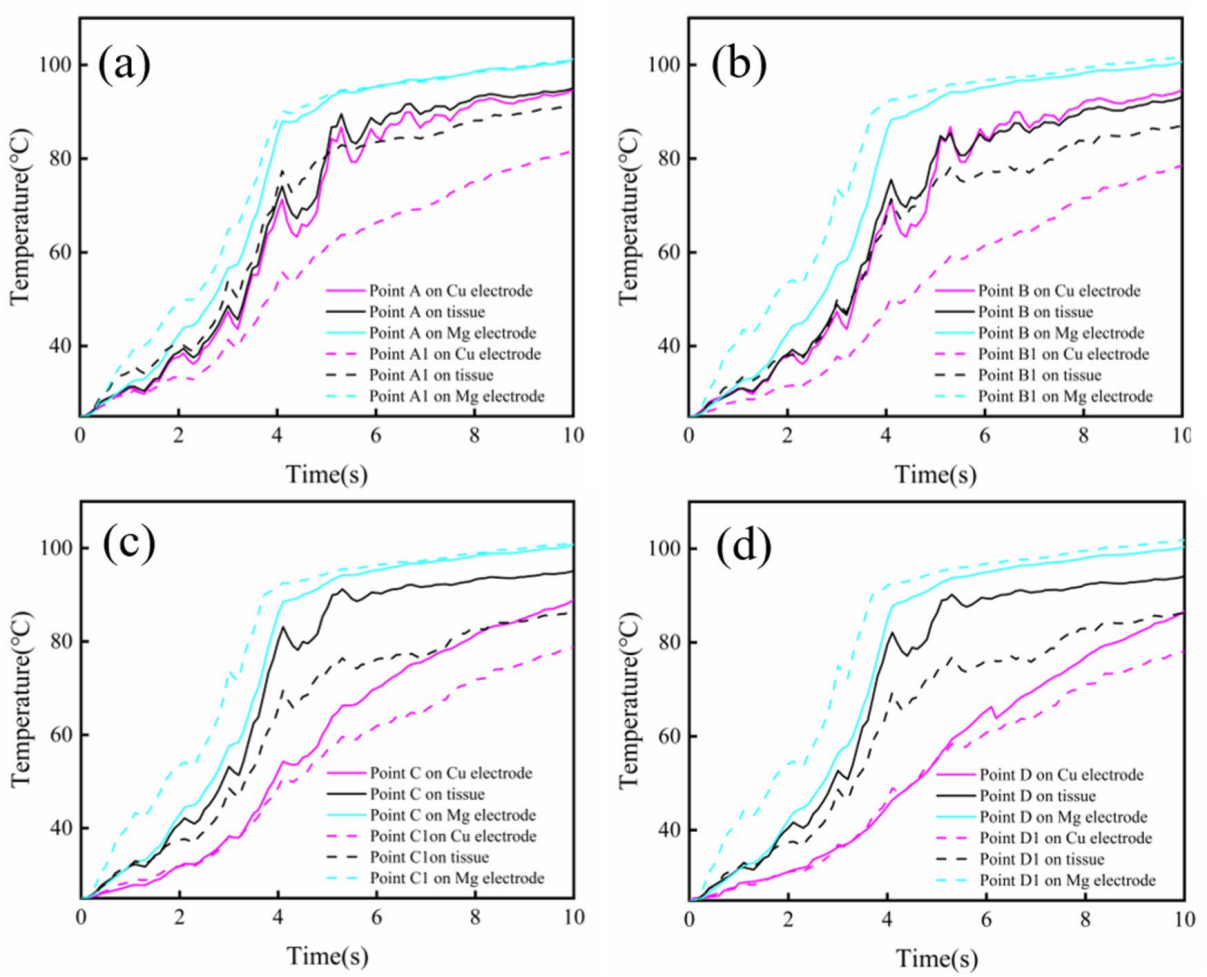
Temperature variations of different positions corresponding to domain points of (**a**) A-A1, (**b**) B-B1, (**c**) C-C1, (**d**) D-D1.

We further studied the temperature variations of separate domain point on the outer Cu electrode, the welded tissue and the inner Mg-based electrode in the simulations, as shown in Fig. 6. The results suggested that the temperature on the inner Mg-based electrode was higher than that on the intestinal tissue, and the lowest temperature appeared on the outer Cu electrode at the same corresponding domain point. Furthermore, the temperatures on the outer Cu electrode at the corresponding domain points of A and B were similar to those on the welded tissues, whereas the temperatures on the outer Cu electrode at the corresponding domain points of C and D were significantly lower than those on the tissues. On the whole, the Mg-based electrode with structural feature of long ring presented lower welding temperature because of lower heat generation and higher degree of thermal diffusion. These results above indicated that the proper structure design of tissue welding electrode could achieve a reduced welding temperature via controlling heat generation and promoting thermal conduction, and thus improving the quality of tissue welding and ensuring the activity of tissue cells.

### Biomechanical properties of anastomotic stoma

Preliminary experiments of tissue welding ex vivo showed that a compression pressure of 176 kPa ensured sufficient force to fuse tissues and prevented mechanical damage. RF tissue welding results from the combined effects of compression pressure, output power, and welding time. Further experiments of tissue welding ex vivo revealed that the highest output power of 160 W with a welding time of 6 s achieved effective welding, whereas the lowest output power of 120 W showed significant tissue thermal damage after 12 s. Therefore, we selected output powers of 120 W, 140 W, and 160 W, with welding times of 6 s, 8 s, and 10 s, respectively, to optimize the process parameters in this study.

**Figure 7:**
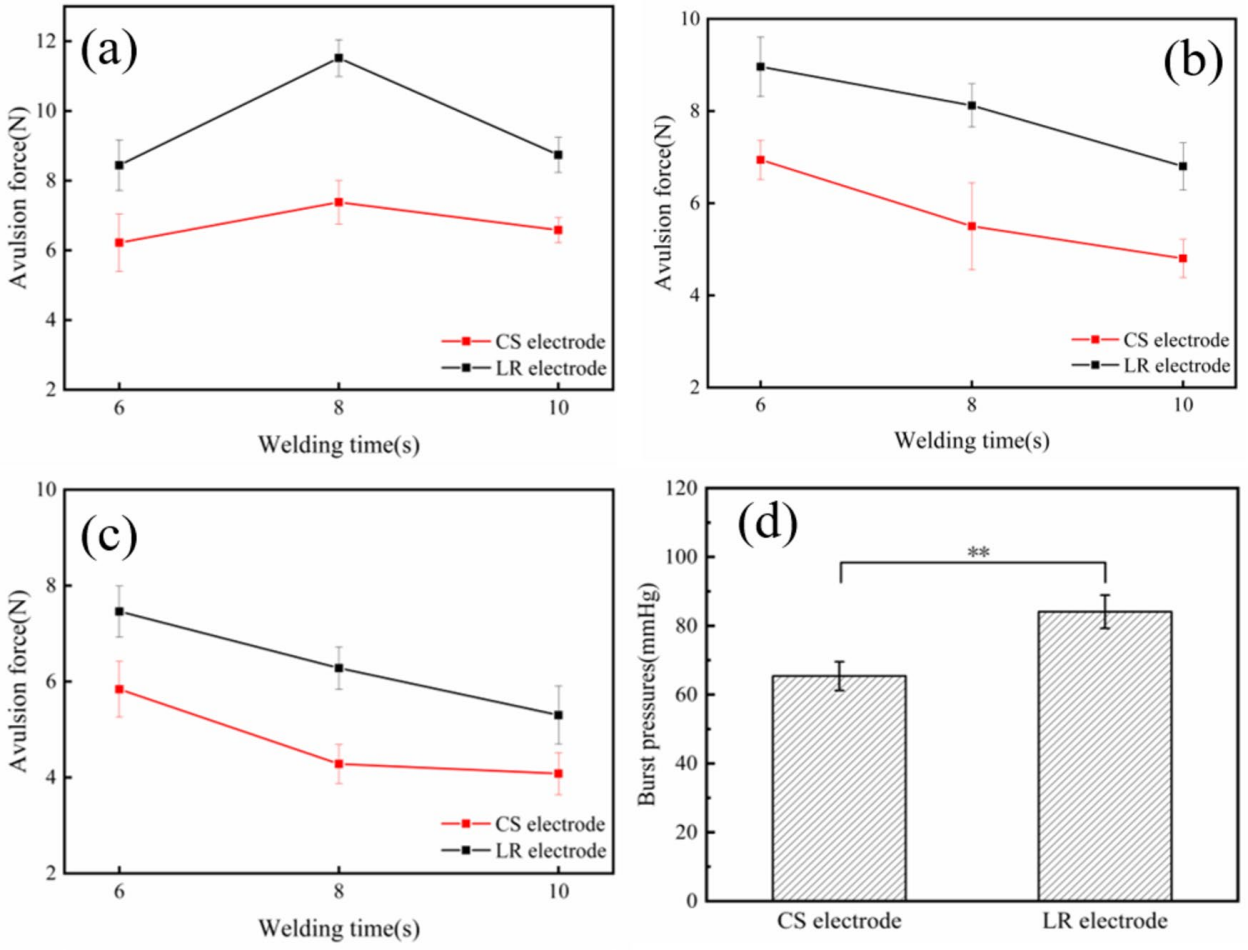
Biomechanical properties of welded tissues under different welding parameters. Anastomotic avulsion force of different welding times on tissues when the applied RF energy was (**a**) 120 W, (**b**) 140 W and (**c**) 160 W, respectively. (**d**) Burst pressures of welded tissues after applying an energy power of 120 W and a compression pressure of 176 kPa for 8 s.

The avulsion force results of welded tissue under different parameters were shown in Fig. 7a–c. It was apparently found that the tissues welded by the LR electrode presented higher anastomotic avulsion force than that welded by the CS electrode under different parameters. Meanwhile, the results also indicated that the highest avulsion forces could be achieved both for the LR (11.52 ± 0.53 N) and CS (7.38 ± 0.62 N) electrode groups after applying an energy power of 120 W and a compression pressure of 176 kPa for 8 s. Besides, the burst pressure (BP) test was conducted to examine the biomechanics of anastomotic tissues under the optimized welding parameters (120 W, 176 kPa, 8 s), and the results presented in Fig. 7d indicated that the burst pressure of anastomotic stoma welded by the LR electrode reached 84.12 ± 4.83 mmHg, which was obviously higher than that welded by the CS electrode (65.41 ± 4.16 mmHg). According to the Mann–Whitney U test, the avulsion forces of the tissue welded by the LR electrode showed significant difference with the P values of 0.003, 0.005, and 0.008 after applying energy powers of 120 W, 140 W, and 160 W, with a constant compression pressure of 176 kPa for 8 s. Furthermore, the Kruskal–Wallis test showed that there was a statistically significant difference in avulsion force and burst pressure between tissues welded by the CS and LR (p < 0.05), under the optimized welding parameters of 120 W, 176 kPa, and 8 s. Overall, results from the avulsion force and burst pressure measurements indicated that the LR electrode possessed obvious advantages to achieve a higher biomechanical property of welded anastomotic stoma, which contributed to improve the safety and effectiveness of RF intestinal tissue welding through applying the novel designed implantable Mg based electrode.

### Temperature variations of welded tissue

**Figure 8:**
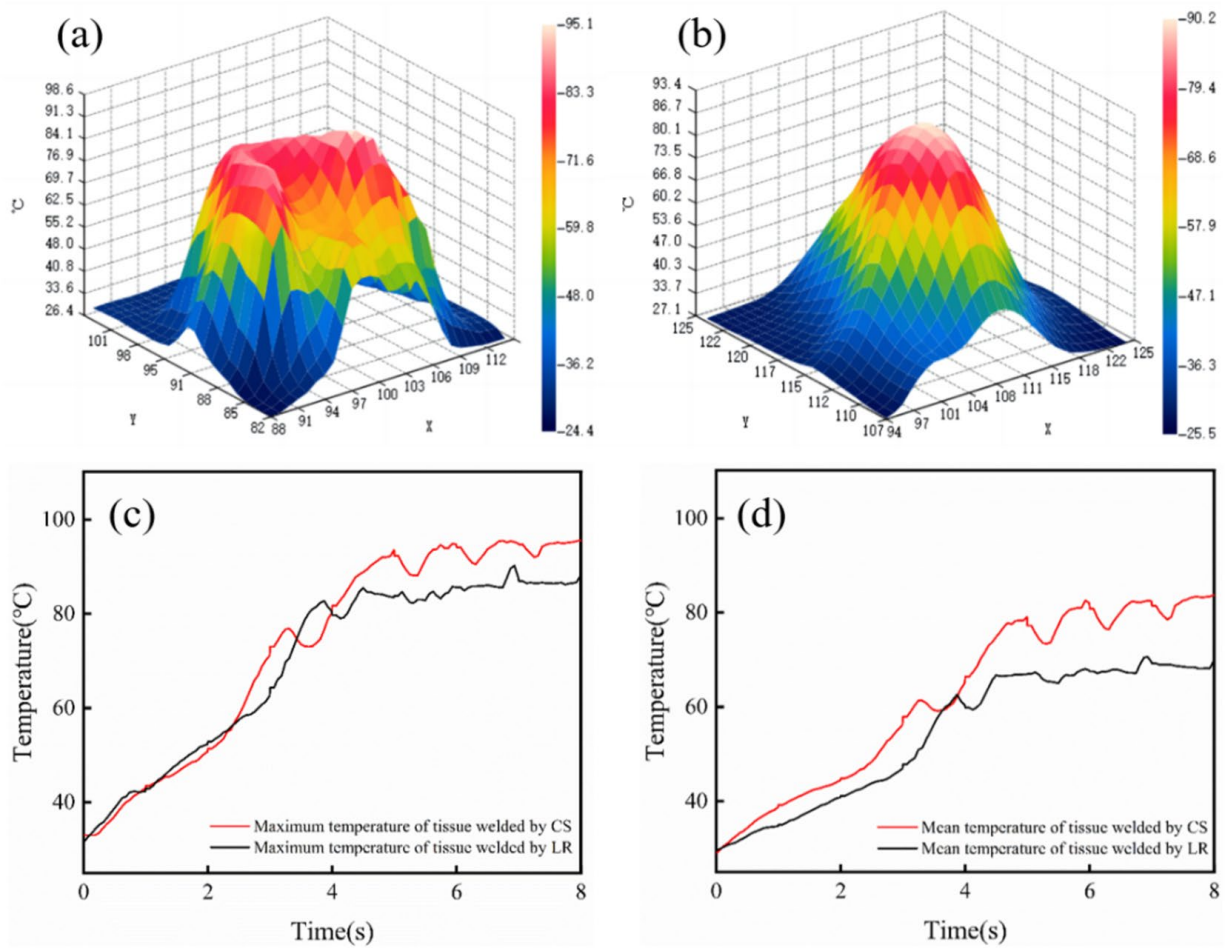
Real-time temperature variations monitored by an infrared imager. 3D thermographs of temperatures for the tissues welded by the CS (**a**) and LR (**b**) electrodes; The maximum (**c**) and mean (**d**) temperature variations of tissues welded by CS and LR electrodes.

In order to investigate the temperature variations of welded tissue, the three-dimensional (3D) temperature distributions on the anastomotic stoma were continuously monitored using an infrared imager, and the results were shown in Fig. 8. It was obviously found that the temperature distributions in both groups showed a symmetrical trend due to the symmetrical structures of the electrodes (Fig. 8a,b). Furthermore, the overall temperatures with a maximum value of 94.8 °C and 90.4 °C were observed for the tissues welded by the CS and LR electrodes, respectively. In addition, it could be found that the mean temperature was 71.3 °C for the tissue welded by the LR electrode, which was significantly lower than that obtained by using the CS electrode (81.4 °C) when the welding time was 8 s (Fig. 8c,d). Therefore, these results confirmed the advantages of the LR electrode over the CS structure in controlling the temperature of anastomotic stoma, which was in favor of reducing the lateral tissue thermal damage during tissue welding process.

### Histopathological examination

**Figure 9:**
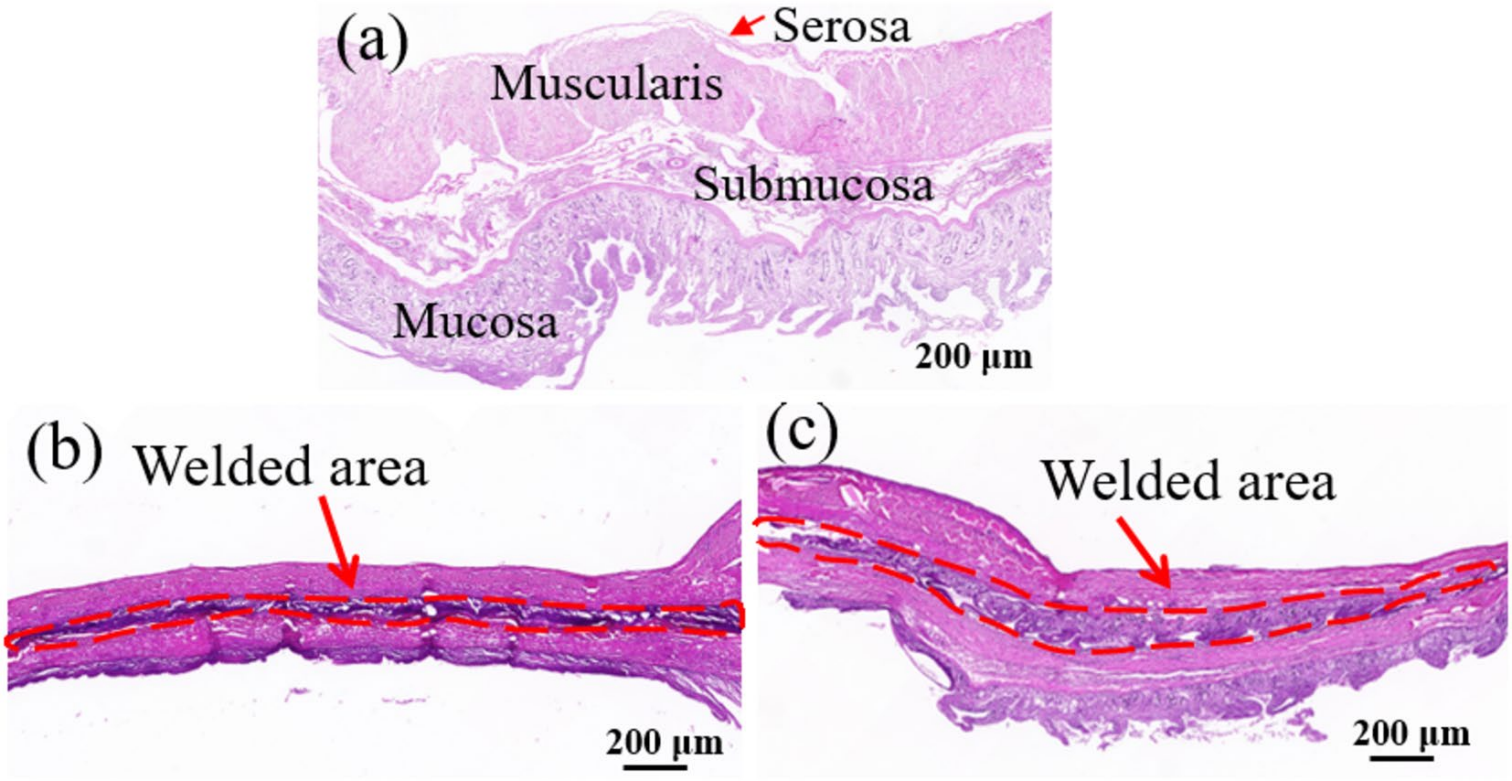
H&E images of tissues. (**a**) Normal intestinal tissue. Anastomotic stoma welded by the CS (**b**) and LR (**c**) electrodes.

The anastomotic stomas were obtained through the tissues welded by the two electrodes under the optimized welding parameters (120 W, 176 kPa and 8 s), and then we carried out hematoxylin–eosin (H&E) staining to examine the histopathological change of the junction of tissues, as shown in Fig. 9. Comparing with normal intestinal tissues, the welded tissues presented tighter structures under the influences of compression pressure and RF energy, with a welding area width of 104 ± 9 μm for the CS group and 150 ± 8 μm for the LR group. The anastomotic stoma welded by the CS electrode (Fig. 9b) showed a super tight junction between the tissues. However, serious tissue dispersion inside the serosal layer existed due to a whole uniform pressure on the welding area exerted by the CS electrode. While for the LR electrode groups, more complete tissue junction was formed under the same welding condition because the LR electrode with partially hollowed-out and partially solid structure could avoid excessive tissue extrusion (Fig. 9c). Therefore, the LR electrode with the hollow structure in the welded area is beneficial to obtain an ideal quality of anastomotic stoma with more compact tissue junction without distinct mechanical damage.

## Discussion

Compared with conventional mechanical anastomosis method, RF tissue welding is an innovative technology for tissue sealing which exploits heat-induced rapid tissue fusion without introducing any foreign bodies^8,9^, and thus, is expected to reduce post-operative complications in existing intestinal anastomosis procedures including rejection, bleeding and leakage. Tissue welding technique is based on a denaturing and cross-linking process of collagen under simultaneously applied RF energy and compression pressure (CP) to form a structural bond between neighboring tissues. However, this technology is challenged by large area thermal damage and low bonding strength, causing significant necrotic tissue at the anastomotic stoma and consequently leading to intestinal fistula. Moreover, removal of conventional metal electrode following completion of tissue welding usually leads to laceration and perforation of the intestine. Therefore, it is necessary to innovate the material and structure of the electrode with the aim of avoiding the retrieval of welding device, reducing tissue thermal damage, improving the bonding strength, and consequently improving surgical safety of RF tissue welding technology for clinical application.

Bioresorbable Mg and its alloys offer unique opportunities to design implantable electrodes that can self-eliminate after performing temporary functions of tissue welding and mechanical support, thereby avoiding a removal operation and improving considerably the patient’s comfort and safety. Combining with outstanding physical and mechanical properties, Mg alloys have attracted much fundamental research and valuable exploration to develop new generation medical devices like bioresorbable vascular stents^26,27^ and orthopedic implants^28,29^. The low corrosion resistance of Mg alloy makes it appropriate candidate for developing an implantable electrode for next-generation RF tissue welding with multi-functions: (1) as an electrode to conduct RF current to complete tissue welding; (2) as a scaffold to support and strengthen the anastomotic stoma to avoid laceration and stenosis during the tissue healing stage; (3) completely dissolved or absorbed without additional surgical intervention, avoiding laceration and perforation of the anastomotic stoma. Previous study demonstrated that the Mg-Nd-Zn-Zr alloy scaffold can provide support in blood vessels for approximately 3–4 months after implantation^19,30^, thereby meeting the time requirements for intestinal tissue recovery.

In this study, we developed an implantable electrode based on bioresorbable Mg-Nd-Zn-Zr alloy with the structural features of cylindrical surface (CS) and continuous long ring (LR) in the welding area for next-generation RF intestinal tissue welding. The effect of different structures on temperature distribution, biomechanical strength and pathologic change of anastomotic stoma under different welding parameters were systematically investigated through finite element analysis and experiment of tissue welding ex vivo, with the purpose of verify the feasibility of implantable electrode based on bioresorbable Mg alloy to improve the safety and effectiveness of tissue welding based on RF energy.

It is well accepted that the structure of electrode determines the outcome of RF tissue welding by means of affecting the process of heat generation and diffusion^31^. This elevated temperature not only ensures the heat required for complete tissue fusion, but also match the temperature that retains certain tissue biological activity to facilitate postoperative tissue repair and healing. The LR electrode with partially hollowed-out and partially solid structure in the welding area could ensure the rise of temperature in the solid area and simultaneously promote heat diffusion in the hollowed-out area. In addition, the tissue in the hollowed-out area was more intact and the temperature sustained lower than that in the solid area, which was beneficial to maintain the viability of tissue. In contrast, the CS electrode, with its cylindrical surface features, made full contact with the tissue and applied RF energy uniformly. This comprehensive increase in the temperature of the welded tissue resulted in more significant thermal damage. FEA results showed that the mean temperatures were 91.8 °C and 82.8 °C for the tissues welded by the CS and LR electrodes when applying an RF energy of 120 W for 10 s, and the corresponding proportions of necrotic tissue for the two structures were 79.6% and 67.9%, respectively. Furthermore, the mean temperatures monitored by an infrared imager during the welding experiments were 81.4 °C and 71.3 °C for the tissues welded by the CS and LR electrodes, respectively. Therefore, results from FEA and experiment of tissue welding ex vivo indicate the advantages of the LR electrode in controlling the welding temperature between 70 and 80 °C, which basically matches the ideal temperature recommended by the reference of^32^ for tissue fusion.

Compression pressure, energy output and welding time are the key parameters to determine the outcome of the RF tissue welding. However, higher compression pressure is easily to overly squeeze the tissue and destroy its integrity, while higher energy power and longer welding time easily lead to thermal necrosis of the anastomotic tissue. On the other hand, lower compression pressure and energy power as well as inadequate welding time can lead to unreliable tissue bonding, which is easy to cause the occurrence of anastomotic fistula. Therefore, the welding parameters need to be explored according to the specific structural characteristics of electrode to achieve a better quality of anastomotic stoma. Burst pressure test and avulsion force measurement are the most common methods used to assess the biomechanical strength of anastomotic stoma in RF tissue welding^33^. Intestinal tissue has its own dynamic performance, including tonic contraction, segmental motility, peristalsis, and peristaltic rush. The frequency and amplitude of contractions in each exercise phase can complete the basic functions of the intestine. Contraction amplitude of segmental motility is around 5–50 mmHg, which is the main mode contractile activity in the intestine^34^. The normal physiological function can be achieved when the avulsion force for the anastomotic stoma reaches 10 N^35^. In this study, the maximum average avulsion forces were 7.38 ± 0.62 N (CS) and 11.52 ± 0.53 N (LR) and the maximum mean bursting pressure were 65.41 ± 4.16 mmHg (CS) and 84.12 ± 4.83 mmHg (LR), respectively, when applying an energy power of 120 W and a compression pressure of 176 kPa for 8 s, indicating that a satisfied quality of anastomotic stoma with improved biomechanical strength could be obtained through applying the implantable Mg-based electrode with continuous long ring structural feature in the welding area. Compared to using the CS electrode, tissue integrity was better preserved within the hollowed-out zones when using the LR electrode, thus avoiding excessive tissue squeezing and potential mechanical damage. Therefore, applying the LR electrode can achieve a higher tissue connection strength. The results can be further confirmed by H&E staining that the histopathological structure of the tissue welded by the LR electrode is more intact compared with that welded by the CS electrode under the optimized welding parameters.

## Conclusions

In this study, an implantable multi-functional electrode based on bioresorbable Mg-Nd-Zn-Zr alloy was designed with the structural features of cylindrical surface (CS) and continuous long ring (LR) in the welding area for next-generation RF tissue welding application. Results from FEA and experiment of tissue welding ex vivo indicated that the optimal design of electrode structure and reasonable selection of welding parameters (120 W, 176 kPa, 8 s) were effective ways to improve the quality of anastomotic stoma by reducing thermal damage and simultaneously improving biomechanical strength. Due to excessive squeezing and the uniform application of RF energy, the tissue welded by the CS electrode exhibited lower strength and more pronounced thermal damage. In contrast, an ideal welding temperature between 70 and 80 °C could be obtained by applying the LR electrode, and the mean avulsion force was 11.52 ± 0.53 N and the mean burst pressure was 84.12 ± 4.83 mmHg, meeting the clinical requirements of tissue anastomotic strength. Therefore, the application of the implantable Mg-based electrode can improve the safety and effectiveness of RF tissue welding technology. However, the studies on implantable electrode based on bioresorbable Mg alloy are still in its infancy and a considerable amount of work is required prior to their applications in animal and clinical trials in terms of material degradation rate and mechanical support.

## Data Availability

The datasets used and/or analysed during the current study available from the corresponding author on reasonable request.
